# Supplementary material for: Intensive Care Outcomes of Patients after High Dose Chemotherapy and Subsequent Autologous Stem Cell Transplantation: A Retrospective, Single Centre Analysis
Source: Cancers (Basel). 2020 Jun 24;12(6):1678. doi: 10.3390/cancers12061678 (PMC7352739; doi:10.3390/cancers12061678)
Supplement: Supplementary file 1 [file cancers-12-01678-s001.pdf]

*Supplementary Materials*

# Intensive Care Outcomes of Patients after High Dose Chemotherapy and Subsequent Autologous Stem Cell Transplantation: A Retrospective, Single Centre Analysis

**Panagiotis Karagiannis, Lena Sänger, Winfried Alsdorf, Katja Weisel, Walter Fiedler, Stefan Kluge, Dominic Wichmann, Carsten Bokemeyer and Valentin Fuhrmann**

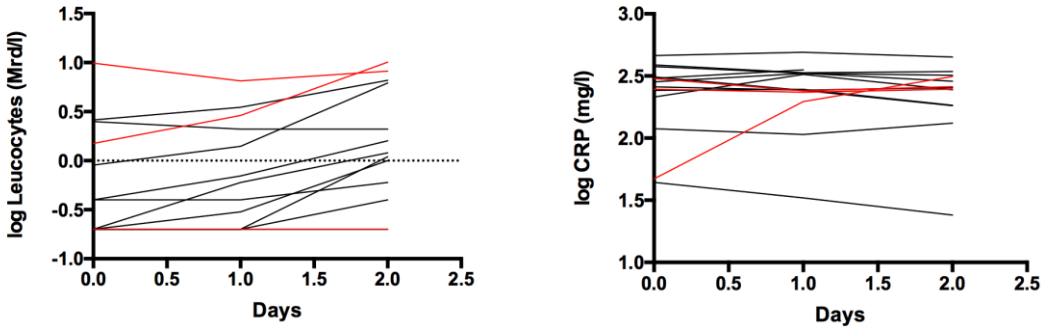


**Figure S1.** Laboratory parameter of patients admitted to the intensive care unit in association with the treatment (cohort 1). Data points include admission and first two days of treatment at the ICU. Red lines depicting deceased patients.

**Table S1.** Depicting the total number and percentage of different high dose chemotherapy regimens.

| Variable *n* (%) | All Patient (*n* = 256) |
|---|---|
| Melphalan | 125 (49) |
| BEAM | 54 (21) |
| PEI | 10 (4) |
| CE | 41 (16) |
| BCNU/Thiotepa | 18 (7) |
| other | 8 (3) |

**Table S2.** ROC curve analysis independent risk factors for ICU admission in relation to their HDT and ASCT treatment.

| Variable | AUC | *p*-Value | Cutoff point | Sensitivity | Specificity |
|---|---|---|---|---|---|
| Duration of Aplasia | 0.75 | 0.001 | 6.5 | 74.3 | 66.7 |
| Charlson Score Sum | 0.70 | 0.005 | 3.5 | 66.8 | 76.5 |

© 2020 by the authors. Licensee MDPI, Basel, Switzerland. This article is an open access article distributed under the terms and conditions of the Creative Commons Attribution (CC BY) license (http://creativecommons.org/licenses/by/4.0/).
